# Ethical care requires pragmatic care research to guide medical practice under uncertainty

**DOI:** 10.1186/s13063-021-05084-0

**Published:** 2021-02-15

**Authors:** Tim E. Darsaut, Jean Raymond

**Affiliations:** 1 Mackenzie Health Sciences Centre, Department of Surgery, Division of Neurosurgery, University of Alberta Hospital, 8440 - 112 Street, Edmonton, Alberta T6G 2B7 Canada; 2 Department of Radiology, Service of Interventional Neuroradiology, Centre Hospitalier de l’Université de Montréal – CHUM, 1000 St-Denis, room D03-5462B, Montreal, QC H2X 0C1 Canada

**Keywords:** Research ethics, Clinical trials, Equipoise, Therapeutic obligation, Medical care, Evidence based medicine

## Abstract

**Background:**

The current research-care separation was introduced to protect patients from explanatory studies designed to gain knowledge for future patients. Care trials are all-inclusive pragmatic trials integrated into medical practice, with no extra tests, risks, or cost, and have been designed to guide practice under uncertainty in the best medical interest of the patient.

**Proposed revision:**

Patients need a distinction between validated care, previously verified to provide better outcomes, and promising but unvalidated care, which may include unnecessary or even harmful interventions. While validated care can be practiced normally, unvalidated care should only be offered within declared pragmatic care research, designed to protect patients from harm. The validated/unvalidated care distinction is normative, necessary to the ethics of medical practice. Care trials, which mark the distinction and allow the tentative use of promising interventions necessarily involve patients, and thus the design and conduct of pragmatic care research must respect the overarching rule of care ethics “to always act in the best medical interest of the patient.” Yet, unvalidated interventions offered in contexts of medical uncertainty cannot be prescribed or practiced as if they were validated care. The medical interests of current patients are best protected when unvalidated practices are restricted to a care trial protocol, with 1:1 random allocation (or “hemi-prescription”) versus previously validated care, to optimize potential benefits and minimize risks for each patient.

**Conclusion:**

Pragmatic trials can regulate medical practice by providing (i) a transparent demarcation between unvalidated and validated care; (ii) norms of medical conduct when using tests and interventions of yet unknown benefits in practice; and eventually (iii) a verdict regarding optimal care.

A central concern of medical care ethics is “What is good medical practice?” Medical tests and interventions must be evaluated and the evaluation of care must be performed within care. Unfortunately, such questions and their answers have been relegated to research, considered a separate domain since the Belmont report [1]. Medical practice now has to wait for the results of optional research to verify or control its activities, but this research is constrained by multiple conceptual, regulatory, and institutional obstacles [2, 3]. The end result is that medical care routinely includes unverifiable practices that can do more harm than good [4–6]. This fundamental problem is pervasive and concerns everyday medical tests and interventions. The main purposes of the present article are to propose a novel analysis that challenges the orthodox research-care separation and to offer a coherent conceptual and ethical framework supporting the necessity of using pragmatic care trial methods to guide good medical practice in the presence of uncertainty [2]. The care trial methodology and examples of its use in practice are detailed elsewhere [2, 7].

The research-care demarcation was meant to identify research subjects and protect them from research conceived as an activity that uses vulnerable individuals as means to obtain “generalizable knowledge” for the sake of Science. Let us call this type of research explanatory research, after Schwartz and Lellouch [8]. But explanatory research is only one type of medical research, and as we will see, not the most pertinent type for patients and clinicians. While explanatory research must be regulated to protect vulnerable patients from being used to gain knowledge, other research methods, such as pragmatic care trials, are designed to protect patients from another threat, the unregulated use of unvalidated tests and interventions within medical care. Care trials also serve to guide medical practice under uncertainty and to eventually define good medical practice [3]. Unfortunately the current research regulation does not distinguish explanatory from pragmatic care research.

The research-care distinction has recently been questioned, with a call to relax some of the constraints on medical research to allow the development of a “learning health care system” [9–11]. To justify this move, seven types of moral obligations borne by all parties, including patients, have been proposed. But we see no need to impose new moral obligations on patients, such as “to contribute to the common purpose of improving the quality and value of clinical care and health care systems” [11]. Much can be achieved by focusing on the care side of the care research distinction. One aim of the present paper is to show that medical care ethics demands that scientific methods be reintroduced within practice as norms of medical conduct. Medical care ethics requires reliable, repeatable, publicly verifiable methods to identify what constitutes good medical practice. In other words, medical ethics needs a practical science to distinguish tests and interventions that improve patient outcomes from those that could be useless or harmful, a distinction that should transparently be revealed to all patients engaging in any diagnostic or therapeutic plan. In the meantime, while good practice has yet to be defined, what should patients and clinicians do? The ethics of medical practice must still guide actions in the care of current patients; to do so, it requires some norms to guide the use of promising tests and interventions until they are validated as beneficial. We have proposed pragmatic care trials integrated to practice to fulfill this need. The Belmont separation between care and research needs to be reassessed to leave room within care for declared care research.

Care research proposes to protect the medical interests of each patient by transparently revealing the uncertainty, by taking the uncertainty seriously, and by acting accordingly, changing practice immediately. Unvalidated interventions, justified by good reasons and intentions, but practiced despite a lack of knowledge of effects on patient outcomes, should not be practiced just the same as validated care, outside any evaluative context. In the proposed framework, opting for an unvalidated intervention requires special precautions to mitigate the risks and optimize chances of a good outcome for each patient, until the uncertainty is lifted, and the best practice is identified. Unvalidated interventions are then only offered as “hemi-prescriptions,” always balanced with a 50% chance of receiving previously validated care.

The revision we propose does not primarily aim to gain knowledge, to improve health care delivery systems for the good of society or for future patients (even though it will eventually provide such results). The emphasis on gaining knowledge is the source of the misunderstanding that has rendered research an apparently extraneous affair in the care of current patients. We grant that if new knowledge that can accrue from medical research has future practical value it is to eventually provide interventions that will improve patient outcomes. But one problem with the current research-care demarcation is the assumption that the sole role of scientific methods in medicine is to decipher or extract new knowledge that can then be applied in the future. One central thesis of this paper is that pragmatic trial methods can *immediately* be put to the service of patients in need of care in the presence of uncertainty. In other words, the value of such methods is not *mediated* by the eventual gain in knowledge. Pragmatic care trials provide a framework that can protect patients from what otherwise would be the unregulated use of potentially harmful interventions, a framework that appropriately changes medical practice immediately, long before new knowledge will eventually be found. In short, scientific methods are essential to good medical practice, especially when knowledge as to what to do is in wont.

What is frequently pertinent to the care of current patients is the *lack of* knowledge, the uncertain value and unknown consequences of medical interventions that, if put to the test, may turn out to be useless or harmful. What is good medical practice in such contexts of uncertainty is our main concern. Medicine can be practiced safely in the presence of pervasive uncertainty when care research is integrated to care. What this means in terms of regulation of practice and professional autonomy is within the domain of political considerations that are beyond the scope of this paper [10]. Whether the current regulation of medical research should be reformed is another question that needs much more work. However, much could be achieved through an improved understanding of the role pragmatic care research can play in the best interest of current patients, without radically changing rules and institutions.

We first outline the structure underlying various care and research activities. We then explain the notion of validated care in medical care ethics. We review the Belmont report’s definitions of research and care and how their separation currently obstructs the practice of outcome-based medical care. We then propose that unvalidated care should be considered research and explain how care research can be designed to be in the patient’s best medical interest. We then propose an ethical framework justifying the pragmatic trials we believe are needed to practice good medicine, replacing the research-care demarcation by the validated-unvalidated care distinction. We discuss how the normative role of care research cannot be replaced with observational studies and how it should be integrated within care. Finally, we will review some of the difficulties involved in integrating care research into medical practice.

## Explanatory, pragmatic, and care research

Various research methods are commonly misunderstood and misused. The relationship between knowledge, action, and ends differs depending on the context. Let us start by contrasting care and research. The clinician (agent) prescribes an intervention (action) known to improve the outcome of the patient (end). This, as we will see, is validated care. What if the clinician does not really know what to do? Can research help? In other words, what action can be taken in the light of uncertainty, without changing the end (to improve the outcome of the patient)?

We will discuss observational studies in section “Normative care research cannot be replaced by observations of unvalidated care” the special case where the researcher is an external observer of the behavior of medical practitioners, who have now become research subjects in an observational study [12].

We now introduce the contrast between explanatory and pragmatic research. A researcher (agent) can perform an experiment (action) in the laboratory in order to explain some phenomenon (and gain knowledge). One example of inappropriate laboratory type of research is the Willowbrook hepatitis study, where live virus was intentionally administered to residents to learn about the development of infection [13]. The Willowbrook researchers designed an experiment in controlled conditions to look for a mechanism to gain knowledge. Nowadays, experts recognize a continuum of trial designs along the explanatory-pragmatic spectrum, but for the sake of clarity and simplicity, we will set up the contrast between “purely explanatory” and “purely pragmatic” trials [14]. While best to identify or prove a mechanism, explanatory trials are inappropriate in medical practice for two reasons. First, since patients, clinicians, and study contexts are selected to look for ideal circumstances that could reveal causal signals that might not have been apparent under normal clinical conditions, for example by imposing rigid research protocols on carefully selected patients, the results of such trials do not generally apply in clinical practice [15]. Explanatory trials may also overestimate benefits and underestimate harm [16]. Second, and more importantly, explanatory research loses track of the vital end of the clinician: the goal has been switched from improving patient outcome to “gaining knowledge.” Explanatory trials primarily aim to gain knowledge and advance science [17]. We can now contrast explanatory and pragmatic trials: pragmatic trials are designed to evaluate care interventions flexibly performed by a diversity of clinicians in a diversity of patients in real-world conditions. This solves the first problem of explanatory trials: their applicability to clinical practice. Finally, care trials are specific pragmatic trials (at the pragmatic end of the spectrum) designed to care for patients in the presence of uncertainty; each item of the design of a care trial is chosen to be in the best medical interest of the patient. This addresses the second problem. The clinician is not a foreign observer of phenomena that require explanation, nor a laboratory scientist experimenting with patients for the sake of knowledge; the clinician is a responsible agent that uses scientific methods as means to improve patient outcomes immediately, in spite of the current lack of knowledge.

## The notion of validated care in care ethics

Patients need some norm whereby tests and interventions can qualify as admissible within medical care. In other words, patients need outcome-based medical care. Tests and interventions that have been proven beneficial can then be prescribed as normal or validated care. Unvalidated care, or practicing outside that safe boundary is possible, but it should not be offered in the same manner, even if such tests and interventions seem promising, because they may turn out to be useless or harmful in practice. The ethical way to practice medicine outside normal care is care research, using pragmatic trial methods designed to regulate the unvalidated practice in the best interest of current patients. This self-regulation of unvalidated care is needed until the uncertainty is lifted and the best action is validated by the very trial being proposed [2]. Promising unvalidated tests and interventions can thus eventually be validated and practiced normally or alternatively be abandoned if shown to be harmful through openly declared care research. Since no one knows whether unvalidated care does good or harm as compared to the validated care it intends to replace, pragmatic care trials are at the same time the ethical and scientific way of offering promising but unvalidated care to the individual patient, and the way to eventually identify what constitutes good medical practice. Validated medical care is improvable, open to revision, and can be continuously challenged by rival or innovative options. The verdict regarding which action is best must take into account what is most valuable for most patients, but while the verdict may only come in the future, in the meantime the medical interests of current patients are best protected when unvalidated practices are restricted to an openly declared care trial protocol [2, 3].

## The research-care separation

The current care research demarcation leaves to be desired because it deprives medical practice of scientific methods, inadvertently separates the provision and the evaluation of care, and misses the crucial role trial methods can play in regulating medical practice in the best interest of current patients. We must examine where things went wrong.

The root of the problem can be traced back to past episodes of research misconduct which called for controlling measures [1]. Many problems came with the hasty solution that Belmont commissioners proposed to attempt to regulate medical research while supposedly leaving care untouched, for “fear of opposition from organized medicine” [10].

Problems and assumptions that need to be re-examined include (1) definitions of research and care based on intentions; (2) a failure to distinguish explanatory research designed to gain theoretical knowledge from care research conceived as a way to regulate practice in the presence of uncertainty, and consequently (3) a failure to foresee that by divorcing science from care and constraining all research enterprises without distinction clinicians would be encouraged to act despite the lack of clinical evidence their actions are beneficial or harmful, without the methods necessary to protect patients from unvalidated care.

First, to regulate research but not care required a demarcation between the two. The demarcation of the Belmont report was constructed on *purpose* or *intentions*: Research was defined as “an activity *designed to* test a hypothesis, permit conclusions to be drawn, and thereby to develop or contribute to generalizable knowledge” [1]. This is the formal scientific testing methodology, now reserved for research. This is already worrisome, but the separation also divorced the ethics of medical practice from the reality of patient outcomes.

In Belmont, “practice” referred “to interventions *designed* solely to enhance the well-being of an individual patient and that have a reasonable expectation of success” [1]. With such a vague definition a broad range of practices are permissible, and almost any promising experimental, unvalidated test and interventions can be considered care, as shown in the following sentence from the Belmont report: “When a clinician departs in a significant way from standard or accepted practice, the innovation does not, in and of itself, constitute research. The fact that a procedure is “experimental,” in the sense of new, untested or different, does not automatically place it in the category of research” [1].

The current regulation emphasizes: “If there is any element of research in an activity, then that activity should undergo review” [1]. However, on the side of practice, experimental tests and interventions are considered “care” without the need for review, “provided no conclusion can be drawn” [1]. Thus this demarcation encourages doctors to use unvalidated interventions within the care context. Patients and medical practice do need a demarcation—not one that relies solely on intentions or purpose, but on the *results* of care. Otherwise good medical care, and the central principle of care ethics, “to always work in the patient’s medical interest,” beyond good intentions, cannot be defined (We contrast the Belmont report definitions with our proposal in Table 1).

**Table 1:** Definitions of some care and research terms and concepts

	Current definitions	*Proposed revisions*
Practice	“Interventions designed solely to enhance the well-being of an individual patient and that have a reasonable expectation of success.” [1]	*Practice includes validated care which can be prescribed, and unvalidated care, which is restricted to care research*
Research	“An activity designed to test a hypothesis, permit conclusions to be drawn, and thereby to develop or contribute to generalizable knowledge.”	*Care research guides care in the presence of uncertainty in the best medical interest of current patients*
Boundary in theory	“The general rule is that if there is any element of research in an activity, that activity should undergo review.”	*The crucial distinction is between validated and unvalidated care*
Boundary in practice	Inexistent; experimental interventions can be used as care	*Unvalidated care should only be offered within a care trial*
Experimental interventions	“The fact that a procedure is “experimental,” in the sense of new, untested or different, does not automatically place it in the category of research.” [1, 2]	*Experimental procedures are by definition unvalidated care; they can be offered, but within a care trial.*
Validated care	No definition exists	*Care that has previously been shown to improve patient outcomes in pragmatic trials*
Unvalidated care	Tests and interventions that can and are used in practice but that have never been convincingly shown to improve patient outcomes	*Unvalidated care is promising but experimental care offered within a care trial.*
Unverifiable care	Tests and interventions are practiced in such a fashion that no conclusion regarding their relative merit or resulting patient outcomes can be drawn	*Unverifiable care is not practiced.*
Optimal medical care	Impossible to define	*Optimal care is validated care continuously revised by care research*

The second problem we wish to re-examine is how clinical research is understood. The report reproduces a peculiar conceptual opposition between the particular, associated with individualized care, and the general, associated with research. It is possible to attend to the singularity of an individual patient and yet still admit uncertainty and practice medicine accordingly. If the care of patients should always be individualized, validation that medical actions are actually beneficial requires verification in multiple individuals. It is a dangerous illusion to believe that proper individualized care is possible without a foundation in generalizations reliably shown in multiple individuals, in other words without evidence. This does not mean, when trial methods are introduced to prudently offer experimental test and interventions as promising care, that the goal of the clinician has suddenly changed from providing optimal care to now using patients to gain theoretical knowledge, a change which would presumably require the sacrifice of individualized care for the benefits of Science or of Society. This first assumption has prevailed since Fried, who introduced the concept of 'personal care'. Fried believed, as so many people do, that randomized allocation of treatment options is incompatible with personal care: “The idea of personal care, with its demand for undivided loyalty to the interests of the patient, would thus seem to be violated by this abdication of professional judgment in the interests of the experiment, interests which are not the same as those of the patient in the particular case” [18]. In the presence of serious uncertainty, doctors do not need to abdicate their undivided loyalty to the interests of the patient, nor the use of their professional judgment. What doctors need to abdicate is the notion that the appropriate action can be found without the need for rigorous methodology, and their yet-to-be justified authority to propose unvalidated interventions in the same way as care that has already been validated as beneficial. When clinical judgment and loyalty to the interests of the patient indicate that some validated intervention is in order, then the doctor proposes validated care, as usual. It is only when clinical judgment and loyalty to the patient seem to indicate that an unvalidated test or intervention may be appropriate that the clinician needs to recognize the limits of current knowledge and the dangers of acting single-handedly, outside a well-planned and prudent care research context. This research context does not have to be designed “in the interest of the experiment,” and therefore does not have to “violate the idea of personal care.”

Fried’s second assumption, recently replicated by advocates of the current care research separation [19], is to assimilate all clinical research with laboratory research à la Claude Bernard. Admittedly, many of the research scandals of the twentieth century did use patients as if they were laboratory subjects. But the care research demarcation seems to ignore how pragmatic and particularly how care research differs from such explanatory research. While some explanatory research done in the twentieth century did exploit patients “in the interest of the experiment” as a means to detect causal signals by imposing rigid protocols on selected research subjects in artificial laboratory-like settings, this type of research is clearly not the proper method to use when evaluating medical care in practice. As early as 1967, Schwartz and Lellouch emphasized: “normally, explanatory work must be done on animals, therapeutic trials on human subjects being limited to pragmatic experiments” [8]. Nor is that research methodology the proper way to care for patients in the presence of serious uncertainty. The way randomized allocation of treatment options can be put to the service of individual patients will be discussed in section “The ethical role trial methods can play in the care of patients”.

The last assumption of the Belmont report then follows from the first two. Commissioners did appreciate the importance of clinical research: “Radically new procedures should, however, be made the object of formal research at an early stage in order to determine whether they are safe and effective. Thus, it is the responsibility of medical practice committees, for example, to insist that a major innovation be incorporated into a formal research project.” However, this responsibility was never implemented in reality. The current research-care dichotomy requires medical practitioners to become researchers if they want to verify whether their actions improve patient outcomes: “Research and practice may be carried on together when research is designed to evaluate the safety and efficacy of a therapy. This need not cause any confusion regarding whether or not the activity requires review; the general rule is that if there is any element of research in an activity, that activity should undergo review for the protection of human subjects” [1]. But commissioners did not foresee the consequences of rendering research inaccessible to clinicians in practice. This is a major obstacle to the care research which is essential to be able to practice outcome-based medical care.

Medical uncertainty has for too long been considered a problem that concerns knowledge, the future, and research. What we want to emphasize is that in reality medical uncertainty concerns action, the present patients, and their care. History is replete with examples of medical interventions practiced for decades only to later be shown useless or harmful when properly assessed, as documented in [20]. This is because it is impossible to identify useless or harmful interventions without proper methods. Tests and interventions, practiced on a case by case basis without proof they are beneficial, then proliferate on the assumption that they are in the best interests of patients. Once untested and unverified interventions are admitted and widely practiced as care, trials which assess whether they cause unnecessary morbidity and mortality become difficult if not impossible to conduct [21]. This is the source of the modern problems of overdiagnosis and overtreatment [22].

Although Belmont commissioners did not intend to impact medical care, practical consequences have been immense. Research now comes with separate ethics and regulation and requires additional resources. Confronted with a treatment decision, in the absence of reliable knowledge, clinicians have two options: Option 1 is to remain in the context of care, do their best to choose a course of action to address the clinical dilemma, and act, using unvalidated interventions if need be; Option 2 is to admit the uncertain value of their actions. But then they would need to enter the world of clinical research, along with its own ethics, bureaucracy, regulation, competition, and even its own language: Clinicians are no longer care providers, but “investigators” that “enroll participants,” rather than doctors dedicated to optimize the care of their patients. The research path requires writing grant applications, competing for financial support, submitting projects to evaluations by Ethics Committees, assuring contracts between institutions etc.…This is a complex and fastidious process that needs training and expertise, and with little chance of success [21]. With ever-proliferating bureaucratic hurdles, clinical trials could become the privileged territory of powerful organizations, such as the Industry or public health initiatives, which may be working on their own priorities [23]. The way to offer prudent care in the presence of uncertainty, eventually needed to find out what makes a “good medical practice,” belongs to medical care ethics. It should be simplified and returned within reach of patients and everyday clinicians.

## The demarcation patients need: unvalidated care is research

An ethical framework that requires patients to contribute to a common good such as the improvement of medical knowledge or health care delivery is possible [11]. However, a more urgent task is to return the responsibility for doctors to limit their interventions to those in their patients’ best interests into the ethics of medical practice. As we have seen, separating care and research encourages unverifiable medical care on the one hand and research treating patients as a means to gain knowledge on the other hand (Table 2). Vulnerable individuals do not need to be split into research participants and patients in need of care, no more than clinicians need to be separated between practitioners and investigators. In reality one patient and one doctor are commonly confronted with a medical problem for which no one really knows what to do. This context must be called “research.” Care research must be admitted within medical care if we want to protect patients from practices that may turn out to be harmful. We must learn from the past. In the NSABP study, for example, it was the conventional mastectomy, long practiced as the “safer option” in millions of women which turned out to be harmful, while the less disfiguring experimental treatment proved best [24].

**Table 2:** Assumptions underlying the present research-care dichotomy, contrasted with proposed care research

	Care	Research	*Care research*
Domain	About medical practice	About research	*About medical practice*
Time frame	About particular problems	About future knowledge	*About acting now in the presence of uncertainty*
Concerns	Concerns individuals	Concerns diseases or populations	*Concerns individuals*
Target beneficiaries	Serves current patients	Serves future patients	*Serves primarily current patients, and secondarily future patients*
Treatment protocols	No protocol; care varies with clinical judgment and patient preferences	Rigid protocols to minimize variations	*Flexible protocols adapted to individualized care, in search of verifiable outcomes*
Necessity	A necessity for all patients	Extraneous to care	*A necessity for employing unvalidated tests and interventions*
End results	Unverifiable care	Theoretical knowledge	*Verifiable outcome-based care*
Impact on care	Usual care	Imposes research in the interest of others	*Regulates care in the interest of current patients*

When patients are proposed a certain course of action, they must know if it has previously been validated as beneficial. If not, patients must not only be informed, but somehow protected from unvalidated care. The way to do this is to provide autonomous rules that should be followed when the use of unvalidated care is contemplated. Best care possible is either validated care or care research: care that is being provisionally and tentatively offered at the same time it is being evaluated. In this program, trial methods play an essential role in protecting all patients *within* medical care.

## The ethical role trial methods can play in the care of patients

Admittedly, the role trial methods can play in optimizing medical care before results become available is difficult to understand and randomized allocation of treatment options is poorly accepted in the medical community. When not divorced from the aim of best caring for patients, trial methods such as randomized allocation are crucial to regulate actions, prevent error and morbidity, and balance risks. They may work in providing reliable knowledge in the future, to learn at one point if a treatment is valuable or not, but that will come only after the medical action, regulated in the right way, has been repeated often enough to lift the uncertainty. However, long before an answer becomes available, trial methods immediately impact on medical practice, and rightly so: they are essential to constrain unvalidated actions, to protect current patients from jumping to conclusions, from decisions triggered by unreliable information, biased data, or based on values falsely associated with one option or another. As such some research methods are norms integral to the ethics of good medical care in the presence of uncertainty. Opposing personal care and randomized allocation, as Fried did, is misguided. It is possible to attend to the singularity of each patient and still opt for care trial participation in the best medical interest of the patient, for the reasons for choosing one treatment or the other outside the trial context may be erroneous. We have explained elsewhere how clinical experience, outside randomized trials, can be misleading, for it relies on comparing diverse patients managed using the same treatment (the wrong-axis comparison) [25]. Observing that old patients treated by carotid endarterectomy had twice as many complications as young patients, we were misled for a decade into treating them with carotid stenting. Trials later showed that outcomes were worse with stenting [26]. Randomized allocation and blinding are in effect scientific devices designed to prevent clinicians and patients from acting on potentially erroneous reasons to act.

Let us examine our example in more depth. The NSABP study was not only successful in generating reliable knowledge of significant human benefit: A disfiguring surgical intervention, performed for decades, was found not necessary [24]. This alone should speak in favor of trials being essential to the ethics of a good practice. But finding a final answer to what the good practice is may not be their most important function. What should be emphasized is that, long before the verdict became available, the trial impacted clinical practice immediately, by controlling the use of unvalidated treatments: from a community point of view, the trial prevented many patients from having the standard (but yet-to be known as harmful) total mastectomy. Even more crucial is that at the individual patient level, and in contrast to the innumerable other women treated outside the trial, participation gave each patient a *chance* to escape unnecessary morbidity [24].

It is admittedly unlikely that our explanations will render randomized allocation a welcome entity for the medical community. But this should not count against the rule we propose, for if randomized allocation detracts patients and clinicians from using unvalidated care, it is for the best. To see this, let us examine the alternative.

## Normative care research cannot be replaced by observations of unvalidated care

Admittedly, randomized trials are difficult endeavors [27, 28]. To circumvent research obstacles, an increasingly used proposal is to replace trials with observational studies of large data bases. Some even consider this option as the most pragmatic of pragmatic approaches. Patients and physicians remain in the care context, choosing treatment options according to their preferences. Demographic data, patient characteristics, treatments, and outcomes are collected as variables and exposures for later analyses. This approach, observing medical practices from an outsider’s view, entirely misses the normative role care research can play in controlling unvalidated care and in optimizing patient outcomes in real time. If we are looking for ethical guidance of our actions under uncertainty, the solution cannot be to evade the questioning, and to act as if we knew, waiting for statistical studies to show if we were right or wrong. It is too late to regulate unvalidated care performed on a large scale [29]. If there is a role for medical ethics to play in practice, it must be in guiding conduct, in regulating actions, especially when no one really knows what to do. True, trial methods must affect the way care is normally provided, but that is precisely how they control unvalidated care in the best medical interest of patients.

## Care research as optimal care in the presence of uncertainty

The fundamental principle we propose is that physicians should provide validated care and reserve the use of unvalidated tests and interventions within declared care research [3].

This principle, in the spirit of the classical *primum non nocere* clause, has a number of consequences that remain to be studied in more detail by a new program.

Research ethics must re-examine its pertinence in this context, and such notions as the therapeutic obligation [30], the therapeutic misconception [31] and equipoise [2, 32]. It is not the research methodology, but unvalidated interventions that can harm patients in this context. A therapeutic obligation to use interventions that could be shown harmful if they were properly appraised cannot hold. Concerns regarding therapeutic misconception (the notion that patients think they are being cared for, while they are being subjected to nontherapeutic research) are misplaced, because care research remains care, even if unvalidated care is rightly deprived of the presumption of beneficence and of the medical authority that should be reserved for validated care [33]. Equipoise (conceived as a *condition* to trial participation) should be replaced, in the case of care trials, by the notion that there must be good reasons to exempt clinicians from the general obligation of using unvalidated interventions only within the prudent context of declared care research. If care trials are designed as optimal care in the presence of uncertainty, they should not be obstructed by competition and bureaucracy or be conditioned on winning grants or funding streams, like other research endeavors [3]. They should be encouraged and promoted at all levels. We have previously provided criteria to allow the timely identification and approval of such trials by institutional committees [2].

## Care research is a work in progress

Although our main task was to offer a coherent framework to re-introduce scientific methods within medical care ethics and practice, care research is still in its infancy and we must briefly mention some of the difficulties that remain to be addressed.

While we emphasized the contrast between the primary aims of explanatory and care research (to gain knowledge versus to care for patients in contexts of uncertainty), we do recognize that explanatory research will always play an important role in determining theoretical or mechanistic understanding of diseases and therapy. Yet, one should be careful not to confuse the search for the proof of a mechanism, and the desire to show treatment in a good light [25]. In this work, we have polarized the distinction between explanatory and pragmatic trials; in actuality, explanatory and pragmatic features often coexist within the same trial. Helpful analytical tools have been designed to identify these features and graphically summarize them (PRECIS-2) [14]. To classify trials and identify those that qualify as “pragmatic” remains difficult [34].

There are innumerable ways that pragmatic trials could be integrated into health care. The all-inclusive pragmatic approach could address some long-standing problems, such as examining outcomes for individuals that have previously been automatically excluded from medical research, such as pregnant women [35].

The pragmatic approach has been criticized, and a discussion of risks is merited. The idea that trial participation could be in the best medical interest of the patient is not new [36, 37]. However, the trial should be designed to ensure that the care options that will be randomly allocated have adequately balanced benefits and risks for individuals [2]. The way to balance risks and benefits may be relatively straightforward, for example when the main purpose of the trial is to introduce a promising innovation [25, 38]. The assumption that a well-designed trial constitutes an improvement for individuals compared to unregulated, unvalidated case-by-case clinical decisions remains counter-intuitive to many [39]. This long-standing debate has resurfaced in recent controversies concerning comparative effectiveness trials on the use of supplemental oxygen in neonates and in other pragmatic trials [39–43]. This is no place to discuss these complex issues in detail, but the treatment groups and allocation method must be carefully tailored to each clinical problem, to ensure that patients are still offered optimal care. The use of other recent methods, such as cluster pragmatic trials, also raises ethical issues regarding consent [44, 45]. Consent issues in cluster trials and in research regarding neonates, unconscious or demented patients, are certainly important but beyond the scope of this article.

The prospect of widely accepted trial methods integrated into medical practice may still be a distant goal. In the meantime, we believe that care trials, where each item of a pragmatic trial design is reviewed and selected in the best medical interest of the participating patient, are a prudent first step that should reassure all stakeholders, including participating clinicians and most importantly the patients whose trust we must preserve (see below) [2]. It is heartening that the alleged risks involved in caring for patients within a care research framework can be mitigated because patients and clinicians can always overturn the randomized allocation and return to an individualized decision should they so choose. However, this outlet, or safety valve, is often considered a breach in scientific propriety. It contributes to the idea that pragmatic and care trials trade trial validity for generalizability [46, 47]. Thus, we must now mention some of the scientific concerns that have been brought forward, including lack of blinding, higher rates of non-adherence to allocated treatments, and losses to follow-up and feasibility [47]. Some of these concerns come from trying to analyze pragmatic trials from an explanatory perspective [47, 48], but others remain real problems intrinsic to the pragmatic approach, that are also beyond the scope of this paper.

We now focus on problems we have been confronted with when introducing care trials in practice. Nine care trials are currently being used to guide the care of neurovascular patients in the presence of serious uncertainty. A progress report has been published to explain how the methodology can be used in practice [7]. The integration of trial methods into medical practice requires adjustments on both the research and care fronts. On the one hand, the trial design is adapted to offer care in the best interest of each patient; on the other hand, clinical practice is disciplined to acknowledge the current uncertainty and act accordingly. Unsurprisingly, care trials have raised concerns from both sides: the design adapted to care has been criticized by conventional trialists, and the randomized allocation that protects patients from unvalidated interventions remains poorly accepted by the clinical community. Consequently, on the research side, most care trials we have designed have been declined by funding institutions and their publication has been difficult [48]. On the care side, without financial compensation few centers have participated. Frequent criticisms concerned “informativeness” and feasibility [17, 49, 50].

The idea that trials are solely designed to gain information has unforeseen yet untoward effects. Research agencies and Industry fund research to gain information. The best way to ensure trials will be “informative” is to limit trials to research questions that are easy to answer, but that may not necessarily be pertinent or generalizable to practice. The pressure to deliver information promotes the selection of patients, centers, and outcome measures most likely to maximize the “signal to noise ratio,” in other words explanatory trials, even though they are not appropriate for informing practice as we have seen. The priority on gaining knowledge also leads to the trap of trial feasibility often listed as a necessary condition of informative trials. The idea that patients participate in trials to help advance medical science and that trials at risk of being “uninformative” should be rejected because “preventable uninformativeness is a serious breach of trust and a violation of research ethics” is not confirmed in reality [17]. Most patients participate in trials because they cannot help but assume that trial participation is in their own interest, even when it is transparently disclosed that this is not the case [31]. Thus, it is a duty for clinicians to design and participate in trials that are primarily conceived in the patient’s best medical interests. Getting information must be a secondary goal. The clinical trials clinicians propose should focus on a more important primary goal: to minimize harm related to uncertainty and guide care interventions in real time. Clinicians design and participate in trials, not primarily to advance Science, but because they have to provide optimal care even when no one really knows what to do.

## Integrating care research into practice

**Fig. 1:**
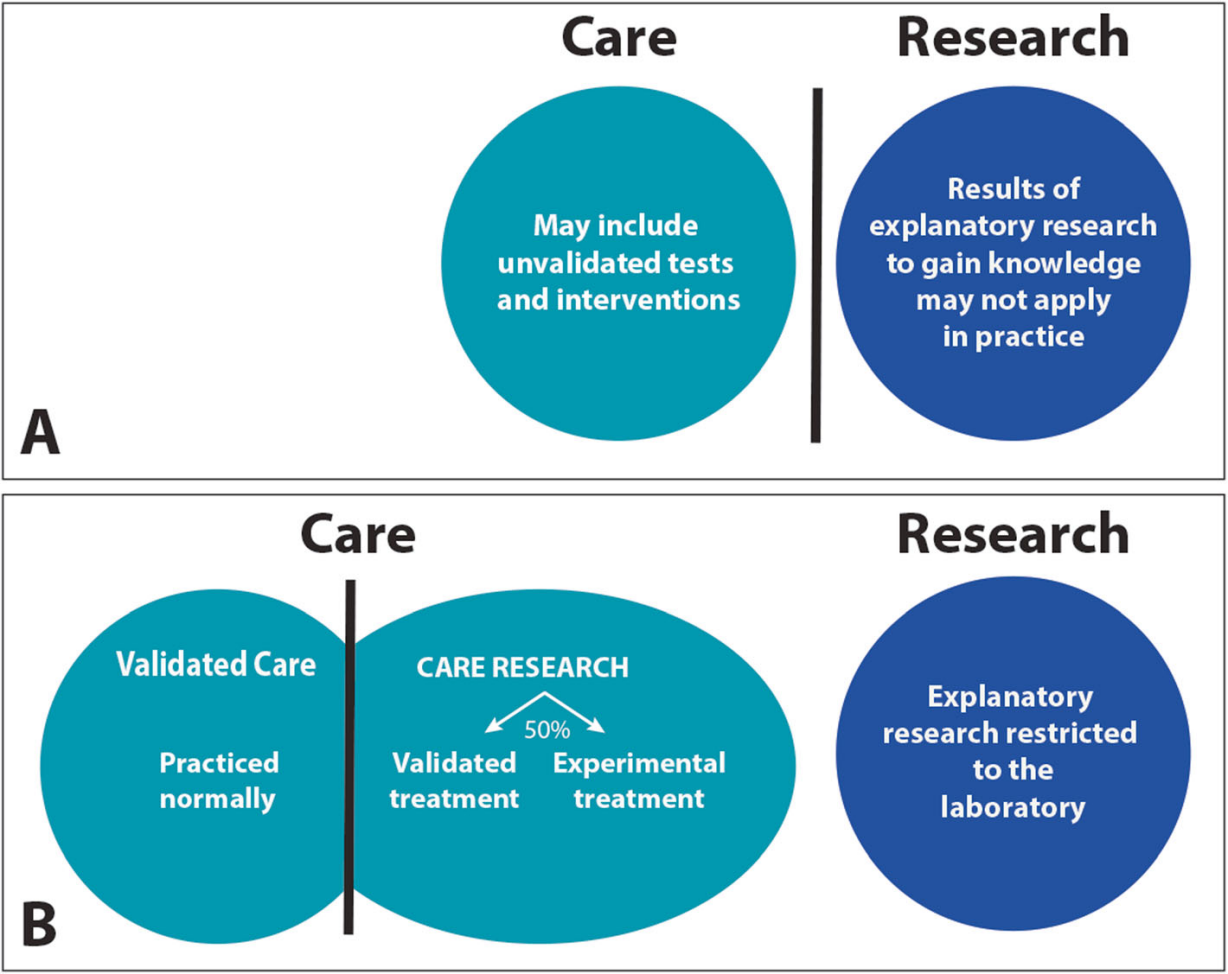
Reconstructing the care research demarcation. **a** According to the prevailing view, research must be cleanly separated from care. Consequently, unvalidated tests and interventions are admitted as care, and explanatory research may use patients as research subjects. In the new program (**b**), optimal care includes validated (but revisable) care and pragmatic care research, but unvalidated tests and interventions are only offered within care research

Optimal medical care, then, is verifiable care that includes two articulated contexts: normal practice and care research. In normal practice tests and interventions that have been shown beneficial can be prescribed. When care is suboptimal, and there is hope for patients to benefit from a promising revision, care research may be indicated, to balance unknown risks and benefits of substituting unvalidated for validated care and to provide rules of proper conduct to protect patients from false promises. When should revisions be integrated into normal practice? When they have been shown to improve outcomes. The research-care distinction must be revised to recognize the validated/unvalidated care distinction (Fig. 1), and the essential role pragmatic care research can play in protecting patients from unverifiable care.

## Conclusion

Optimal medical care continuously needs evaluation and revision. Some trials have ethical functions: They protect the best interests of individuals by regulating the use of unvalidated actions within medical care. These clinical activities can eventually be adopted as normal care or abandoned as useless or harmful. Verifiable medicine can be practiced when care and care research are conjugated.

## Data Availability

Data sharing is not applicable to this article as no datasets were generated or analyzed during the current study.
